# The role of interleukin-17 inhibition in systemic lupus erythematosus—paradoxical hindrance or new therapeutic potential? Results from a systematic literature review and mendelian randomization

**DOI:** 10.3389/fimmu.2026.1706949

**Published:** 2026-03-16

**Authors:** Deepak Nagra, Benjamin Zuckerman, Joy Odia, Samir Patel, Melissa Ong, Maryam Adas, Zijing Yang, Katie Bechman, Mark Russell, Sijan Bhandari, Chamith Rosa, Siwalik Banerjee, Tim Blake, Nicola Gullick, Ganesh Kasavkar, Chris Wincup

**Affiliations:** 1 Department of Rheumatology, University Hospitals Coventry and Warwickshire, Coventry, United Kingdom; 2 Centre for Rheumatic Disease, King’s College London, London, United Kingdom

**Keywords:** IL-17 inhibitor, mendelian randomisation, Paradoxica, Secukinumab, Systemic Lupus Erytheamous

## Abstract

**Introduction:**

Systemic lupus erythematosus (SLE) is a disease with few licensed drugs when compared to rheumatoid arthritis, axial spondyloarthropathies, and psoriatic arthritis. We report on results of a systematic literature review of IL-17i in SLE with appraisal of published cases of both efficacy of treatment and the risk of developing new SLE following treatment with IL-17i through investigation of adverse events in the setting of clinical trials of IL-17i in non-SLE indications.

**Methods:**

We performed a PubMed, EMBASE, and MEDLINE search from inception till 30 June 2025 for case reports of IL-17i–induced SLE. The four EMA-licensed IL-17 inhibitors secukinumab, bimekizumab, brodalumab, and ixekizumab were included for analysis. All four monoclonal antibodies block IL-17A, with bimekizumab having the dual functionality of blocking IL-17A/F. Furthermore, the clinical trial data for secukinumab in psoriasis, psoriatic arthritis, and axial spondylarthritis from inception till 2024 was reviewed for adverse event reporting of new cases of SLE. Both systemic and cutaneous SLE were reported on. We also reported on secukinumab case reports for treating SLE.

**Results:**

Clinical efficacy of IL-17i in case reports of active SLE: in the case of patients treated with secukinumab for known active SLE outside of the clinical trial setting (*n* = 4), the commonest indications for the use of secukinumab were active cutaneous disease and inflammatory arthritis (75%, *n* = 3), with 25% [*n* = 1] having lupus nephritis. All four patients had positive serology for ANA and dsDNA. New-onset SLE following IL-17i in the literature: amongst 56 clinical trials for secukinumab, there have been no reports of drug-induced or paradoxical/new-onset SLE following treatment in the reporting periods for each respective trial (*n* = 13,000). To date, there are no case reports of bimekizumab- or ixekizumab-induced SLE, although there are two case reports for ixekizumab-induced lupus tumidus, which were excluded from the analysis. One case report exists for new-onset SLE in a patient undergoing treatment with brodalumab for psoriasis, and six cases of SLE following initiation of secukinumab were identified. We identified four of the uses of secukinumab in the treatment of SLE.

**Conclusion:**

Despite a handful of case reports for paradoxical reactions with IL-17i, they remain a potential therapeutic option in SLE. All patients recovered upon cessation of the offending IL-17i, and generally patients with drug-induced lupus only require symptomatic management and withdrawal of the offending agent. The efficacy of IL-17i for use in SLE remains unclear due to the limited data from case reports. Among the case reports there was heterogeneity in the reporting of disease activity with no standardization or the use of classic disease metrics such as the SLEDAI or DORIS, which can provide its own challenge in appreciating the results and validating the findings. In conclusion, this is an area that deserves further investigation. Translational research is needed to better understand the role of IL-17 in the pathogenesis of SLE. Dedicated studies and trials of clinical efficacy are needed.

**Systematic Review Registration:**

https://www.crd.york.ac.uk/prospero/, identifier 1338317.

## 1 Introduction

Systemic lupus erythematosus (SLE) is a chronic systemic autoimmune disease defined by anti-nuclear antibody (ANA) positivity, with common manifestations being arthritis, malar rash, nephritis, pleural effusions, and in more severe cases, central nervous system involvement.

Targeted treatments in SLE have primarily focused on B cells through the use of rituximab and, more recently, obinutuzumab; however, additional inflammatory cytokines may be potential therapeutic targets. In comparison to other rheumatic diseases, such as rheumatoid arthritis, axial spondyloarthropathies, and psoriatic arthritis, SLE has few licensed therapies. Given that the pathogenesis of the disease involves abnormal activation of both the innate and adaptive immune responses, there are several potential targets for therapeutic intervention. The development of targeted treatments in SLE has primarily focused on B cells, such as those directed against CD20 (rituximab and obintuzumab) and BAFF inhibition (belimumab). More recently, targeting the type 1 interferon receptor has shown efficacy in active disease, with anifrolumab achieving primary endpoints in clinical trials. However, other cytokine targets, including interleukin-17 (IL-17), remain a possible option to target in SLE.

**Figure 1:**
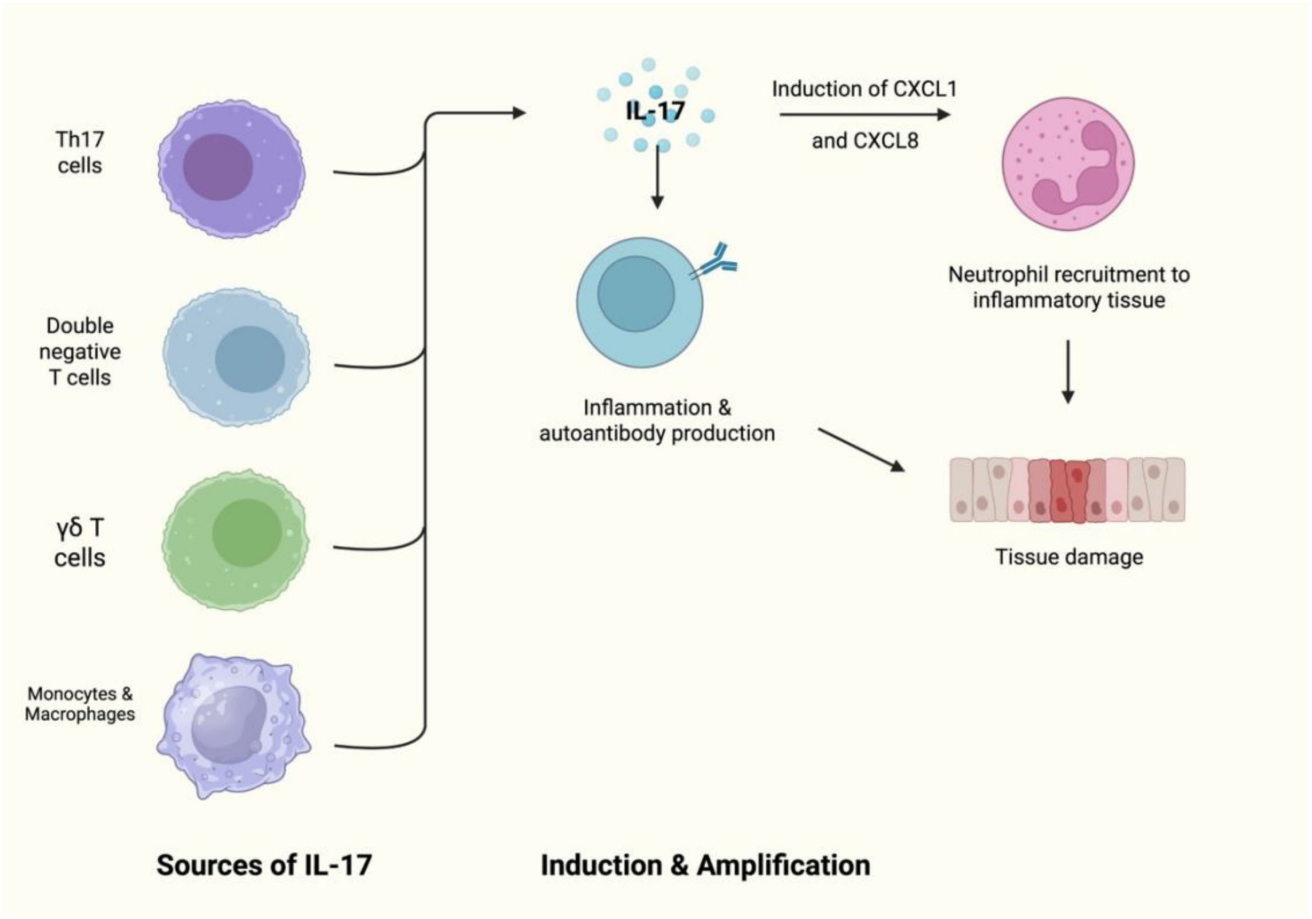
The role of IL-17 in B-cell survival and differentiation.

A number of T-cell subsets produce IL-17, including Th17 cells. IL-17 is a key cytokine involved in autoimmunity and inflammation, with serum IL-17A being increased in those with SLE (1). Furthermore, tissue deposition of IL-17 has been found in kidney and skin biopsy samples in those with SLE (1). IL-17 combined with BAFF is involved in the survival of B cells and their differentiation (2) (**Figure 1**). Inhibition has been explored in SLE with limited success. IL-17 has been proposed as a key mechanism in the pathogenesis of SLE and therefore is an ideal potential target for therapy (2).

The SELUNE trial (NCT05232864) for lupus nephritis was terminated early due to futile results of the interim analysis of the core study. Initially, 275 patients were randomized to receive secukinumab 300 mg or placebo. Amongst the 137 patients receiving secukinumab, only 24% of patients met the primary response with a reduction in proteinuria to under 0.5 mg over 24h, maintaining a glomerular filtration rate of over 60 ml/min, and treatment continuation with no corticosteroid use. In comparison, this was achieved by 36.3% of the placebo group. In a further sub-study analysis of 33 patients, no viable conclusion could be sought on the efficacy of secukinumab (3). In addition, a single trial in discoid lupus was also withdrawn (NCT03866317) (4) without the reason being publicly available. There is limited evidence provided by a small number of case reports describing the use of secukinumab (an IL-17i) in SLE and other systemic autoimmune rheumatic diseases (5).

In parallel paradoxical reactions (onset of disease from a medicine that treats the disease), sarcoidosis and psoriasiform reactions are well-known phenomena amongst IL-17 inhibitors (6, 7). Mechanistically, these reactions are felt to be potentially explained due to epigenetic, genetic (HLA class 2 genes), and T cell–mediated factors (8). Drug-induced lupus, another phenomenon occurring from T-cell disruption, has been widely described for decades. Amongst the monoclonal antibodies, only tumor necrosis factor inhibitors (TNFi) have had an association with drug-induced SLE to date.

There is currently a significant unmet need and major uncertainty relating to the potential role of IL-17 inhibition in the treatment of SLE, and further knowledge is required relating to the potential induction of other immune phenomena in the setting of treatment with drugs that target IL-17, including secukinumab. Herein, we report on the results of a systematic literature review of IL-17i in SLE with an appraisal of published cases of both the efficacy of treatment and the risk of developing new SLE. Further, we evaluated the incidence of new-onset SLE in case reports of patients treated with IL-17 inhibitors for non-SLE indications and also the risk of worsening SLE symptoms in the setting of patients who have been treated with IL-17i for off-label indications.

To date, ixekiuzumab, secukinumab, and bimekizumab are licensed by both the FDA and EMAI for the treatment of psoriasis, psoriatic arthritis, and axial spondylarthritis. Brodalumab is only licensed for psoriasis and psoriatic arthritis.

## 2 Methods

### 2.1 Systematic literature review

#### 2.1.1 Database, sources, eligibility, and data extraction

**Figure 2:**
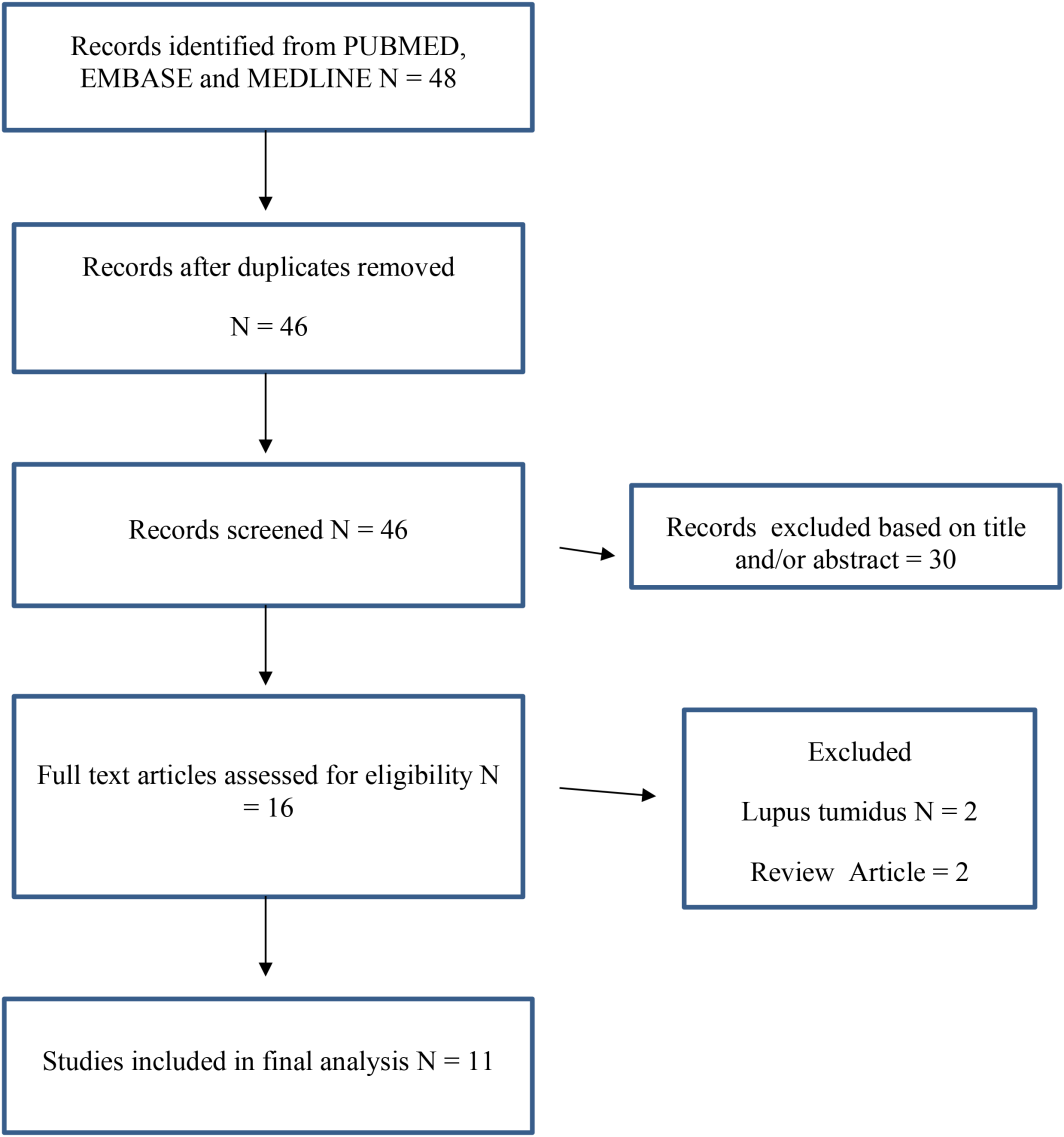
PRISMA.

We performed a PubMed, EMBASE, and MEDLINE search from inception until 30 June 2025 for case reports of IL-17i–induced SLE [PRISMA, **Figure 2** (9)]. The four EMA-licensed IL-17 inhibitors secukinumab, bimekizumab, brodalumab, and ixekizumab were included for analysis. All four monoclonal antibodies block IL-17A, with bimekizumab having the dual functionality of blocking IL-17A/F. The review was registered on PROSPERO (ID 1243558).

The search terms used were secukinumab, bimekizumab, brodalumab, ixekizumab, interleukin-17 inhibitor, IL-17, IL-17 inhibitor, IL-17i, SLE, and lupus.

Furthermore, the clinical trial data for secukinumab in psoriasis, psoriatic arthritis, and axial spondylarthritis from inception until 2024 were reviewed for adverse event reporting of new cases of SLE. Both systemic and cutaneous SLE were reported on. We also reported on case reports of the use of secukinumab in the treatment of SLE (10). Data collection was performed by four reviewers (DN, MA, JO, and MO), and discrepancies were resolved with a fifth reviewer (CW).

#### 2.1.2 Data analysis & selection criteria

Descriptive statistics were used to calculate frequency and percentages amongst the data, with reporting on continuous variables as median and interquartile range (IQR) or mean (SD) as appropriate.

The definitions of paradoxical reaction when compared to drug-induced lupus or new-onset SLE were used as per individual authors from the case reports.

#### 2.1.3 Risk of bias

Two authors (D.N. and B.Z.) independently reviewed the data for risk of bias using the Joanna Briggs Institute (JBI) tool for critical appraisal of case reports & series (11) (**Tables 1**, **2**). The Cochrane risk of bias 2 (ROB2) score was used for randomized controlled trials. Any discrepancies were discussed with a third reviewer (C.W.).

**Table 1:** Summary of case reports of SLE patients treated with IL-17 inhibitor.

SLE patients treated with IL-17i											
Author (Year)	IL-17i	IL-17 indication	Gender	Age	Ethnicity	SLE phenotype	Antibody profile	Biopsy	Exposure length (months)	Management	Notes
Costa 2021 (10)	Secukinumab	Lupus Nephritis class 4	Female	29	Unknown	Arthritis, Raynaud’s, photosensitivity, malar rash, alopecia	Low C3/C4, dsDNA, ANA	Renal	8	Secukinumab. Previous therapy hydroxychloroquine 400mcg, azathioprine, leflunomide, methotrexate, thalidomide, rituximab, mycophenolate, IVIG and belimumab	Complete remission after 8 months of treatment with normalisation of proteinuria.
Dai 2023 (14)	Secukinumab	Psoriasis	Female	23	China	Arthritis	ANA, dsDNA, RNP, rheumatoid factor, Cardiolipin IgG, anti-glycoprotein antibody	Nil	1	Secukinumab	Reduction in dsDNA by 50% and normalisation of CRP and symptoms.
Satoh 2018 (15)	Secukinumab	Psoriasis	Female	62	Japan	Cutaneous, alopecia, anasarca, lupus nephritis	ANA, dsDNA, low C3/C3	Renal and skin	1	Secukinumab 300mg weekly. Previous MMF, prednisolone, cyclophosphamide	Resolution after 45 weeks. Flow cytometry revelated elevate CD38, CCR6 suggesting TH17 involvement. Renal biopsy showed lymphocytic infiltration with IL-17.
Sato 2020 (16)	Secukimumab	Psoriatic arthritis	Male	36	Japan	Arthritis, cutaneous, lupus nephritis	ANA, dsDNA, beta-glycoprotein antibodies		2	Secukinumab 300mg weekly. Previous methotrexate.	Normalisation of CRP and ESR. dsDNA did not normalise.

**Table 2:** Joana Brigs risk of bias for case reports, SLE patients treated with IL-17 inhibitor.

Author ( Year) / JBI Domain	1	2	3	4	5	6	7	8	Overall risk of bias
Costa 2021 (10)	Yes	Yes	Yes	Yes	Yes	Yes	Yes	Yes	Low
Dai 2023 (14)	Yes	Yes	Yes	Yes	Yes	Yes	Yes	Yes	Low
Satoh 2018 (15)	Yes	Yes	Yes	Yes	Yes	Yes	Yes	Yes	Low
Sato 2020 (16)	Yes	Unknown	Yes	Yes	Yes	Yes	Yes	No	Moderate

1) Were patient’s demographic characteristics clearly described? 2) Was the patient’s history clearly described and presented as a timeline? 3) Was the current clinical condition of the patient on presentation clearly described? 4) Were diagnostic tests or assessment methods and the results clearly described? 5) Was the intervention(s) or treatment procedure(s) clearly described? 6) Was the post-intervention clinical condition clearly described? 7) Were adverse events (harms) or unanticipated events identified and described? 8) Does the case report provide takeaway lessons?

#### 2.1.4 Ethics

No ethical approval was required for this project; data were collected from published material. No patient identifiable data were requested or collected.

#### 2.1.5 Mendelian randomization

Two sample Mendelian randomization was used to assess the association between IL-17 proteins and SLE. Genetic associations for SLE were identified from the largest genome-wide association study of 4,036 cases and 6,969 controls (12). Ethical approval had been obtained in all original studies, with relevant citations detailed. Genetic proxies for IL-17 proteins (A, C, D, F, receptor A, and receptor B) were identified from 54,306 participants in the UK Biobank Pharma Proteomics Project (13). Single-nucleotide polymorphisms within 200 kb of each gene, which encodes the relevant protein, were selected, and thresholds of *p*-value < 5 × 10^−6^ and *r*^2^ < 0.1 were incorporated. The strength of the instrument was evaluated through *F*-statistics estimation using beta^2^/standard error^2^ (*F* > 10 suggests minimal weak instrumental bias). We used the two-sample MR Wald ratio, inverse-variance-weighted, to estimate a weighted average of individual variant estimates. Pairwise conditional colocalization was performed to assess genetic confounding through linkage disequilibrium. Bonferroni corrections were applied to account for multiple comparisons. Analyses were performed in R using the *TwoSampleMR* and *coloc* packages.

## 3 Results

### 3.1 Clinical efficacy of IL-17i in case reports of active SLE

In the case of patients treated with secukinumab for known active SLE (10, 14–16) outside of the clinical trial setting (10, 14–16) (*n* = 4), the average age at time of treatment initiation was 32.5 years old (IQR 26–49), and 75% of those treated were female. The most frequent indication for the use of secukinumab was active cutaneous disease and inflammatory arthritis (75%, *n* = 3), with a further case treated for lupus nephritis. All four patients had positive antinuclear antibodies (ANA) and anti-dsDNA antibodies (**Table 3**). One limiting factor amongst these case reports was that response was not defined as either DORIS or Lupus Low Disease Activity State (LLDAS). Response was also not confirmed using conventional validated disease activity scores (e.g., SLEDAI-2K or BILAG) but instead disease inactivity was reported in terms of clinical and biochemical parameters. A median drug exposure time of 6.5 weeks (IQR 5-14) was needed to achieve remission (defined as resolution of symptoms).

**Table 3:** Joana Brigs risk of bias for case reports, paradoxical reactions to IL-17i.

Author (Year)/ JBI Domain	1	2	3	4	5	6	7	8	Overall risk of bias
Ang 2021	Yes	Yes	Yes	No	Yes	No	Yes	Yes	Moderate
Avila-Riberiro 2023 (18)	Yes	No	Yes	Yes	Yes	Yes	Yes	Yes	Low
Chatzimichai 2020 (22)	Yes	No	Yes	No	Yes	No	Yes	Yes	Moderate
Conforti 2020 (21)	Yes	No	Yes	Yes	Yes	Yes	Yes	Yes	Low
Hu 2024	Yes	No	Yes	No	Yes	No	Yes	Yes	Moderate
Kaler 2021 (20)	No	Yes	No	No	No	No	Yes	Yes	High
Louise Koller 2021	Yes	No	Yes	No	Yes	Yes	Yes	Yes	Moderate

The risk of bias amongst these reports was low to moderate, with initial clinical details and serology and histology reports partially missing from the case reports (**Table 1**).

### 3.2 New-onset SLE following IL-17i in the literature

Amongst 56 clinical trials for secukinumab, there have been no reports of drug-induced or new-onset SLE following treatment in the reporting periods for each respective trial (*n* = 12,637). To date, there are no case reports of bimekizumab- or ixekizumab-induced SLE, although two case reports for ixekizumab-induced lupus tumidus were identified and were excluded from this analysis. One case report existed for new-onset SLE in a patient undergoing treatment with brodalumab for psoriasis, and six case reports of SLE following initiation of secukinumab were identified (14, 17–22). The cases are summarized in **Table 2**.

Among those in whom secukinumab and brodalumab were associated with new-onset SLE (*n* = 7), the average age was 57 years (IQR 40–62); 86% were male, and the exposure length was 10 weeks from post-initiation of therapy to the time of onset of symptoms (IQR 8–20). One patient had anti-histone antibodies (six patients did not have anti-histone antibodies present). Among the cases with negative anti-histone antibodies, the diagnosis of SLE was supported through histological findings, a positive ANA or dsDNA, and low complement. Six patients had IL-17i for plaque psoriasis, with one patient for axial spondylarthritis. The most common lupus manifestations were cutaneous lesions and inflammatory arthritis. Biopsies of cutaneous lesions were performed in four patients. The features of the biopsy samples were dermal inflammatory infiltrate of lymphocytes and increased mucoid ground substances. Lupus nephritis was confirmed on renal biopsy in one patient who had proteinuria (5.25 g/24h) with thickened glomeruli and staining of IgG, IgM, IgA, and C3, seen as a full-house pattern in keeping with a class 3 membranous nephritis. In all cases, IL-17i therapy was stopped as part of the management to ensure the offending agent did not cause further clinical deterioration. All patients had resolution of their symptoms.

The risk of bias amongst these case reports was generally low, with only one report scoring moderate risk of bias (Sato, 2020) as there was limited exposure time and historical detail of the patient provided (**Table 4**).

**Table 4:** Summary of case reports with paradoxical reactions to IL-17 inhibitor.

Paradoxical reactions to IL-17i											
Author (Year)	IL-17i	IL-17 indication	Gender	Age	Ethnicity	SLE phenotype	Antibody profile	Biopsy	Exposure length (months)	Management	Notes
Ang 2021 (41)	Brodalumab	Psoriasis	Male	51	Caucasian	Ulcer,rash	ANA, dsDNA, Ro/LA	Skin	5	Discontinuation of Il-17A	
Avila-Riberiro 2023 (18)	Secukinumab	Psoriatic arthritis	Male	68	Unknown	Arthritis, pleural effusion, pulmonary embolus and lupus nephritis	Low c3, ANA, dsDNA	Nil	4	Discontinuation with 0.5mg/kg prednisolone, hydroxychloroquine, enoxaparin, enalapril, mycophenolate	Remission after 33 months
Chatzimichai 2020 (22)	Secukinumab	Psoriasis	Male	39	Caucasian	Cutaneous, arthritis,	ANA	Skin	2	Discontinuation, mometaose furoate	Clinical response after 8 weeks
Conforti 2020 (21)	Secukinumab	Psoriasis	Male	62	Caucasian	Cutaenous	ANA, SSA/Ro, antihistone antibodies	Skin	1	Hydroxychloroquine, topical clobetasol propionate	Clinical improvement after 45 days
Hu 2024 (42)	Secukinumab	Psoriasis	Male	57	Chinese	Lupus nephritis (membranous)	SSA, PM-Scl	Renal	2	Cessation of IL-17A, tacrolimus 1mg od switching to cyclosporine 100mg od and belimumab 600mg with prednisolone 15mg, hydroxychloroquine 200mg.	
Kaler 2021 (20)	Secukinumab	Psoriasis	Female	58	Unknown	Cutaneous	n/a	Skin	8	Cessation of IL7i	
Louise Koller 2021 (43)	Secukinumab	Ankylosing spondylitis	Male	40	Caucasian	Pericardial effusion, arthritis	ANA, dsDNA	n/a	1	15mg prednisolone, cessation of IL-17A. and transition to adalimumab	B27 negative, previous TNFi 24 months prior to IL-17. Remission after 4 months with dsDNA and ANA negative after 15 months

### 3.3 Results of Mendelian randomization

Of the selected proteins, IL-17A did not have a valid protein quantitative trait locus and was excluded from the analysis. The *F*-statistic for all other proteins was > 10. Only genetically predicted IL-17D demonstrated an association with SLE: odds ratio 1.17 per standard deviation increase in IL-17D levels (95% confidence interval [CI]: 1.04–1.32, *p* = 0.007). However, co-localization analyses showed a conditional posterior probability of 6% for a shared causal variant. It is unlikely that the same genetic variant is driving both the increased IL-17D levels and the risk of SLE. This low probability of co-localization suggests that the observed association might be due to genetic confounding rather than a direct causal relationship, meaning that different genetic variants could be influencing IL-17D levels and SLE risk independently.

## 4 Discussion

Herein, we present seven case reports of IL-17i–induced SLE, alongside four cases of SLE managed with IL-17i, supplemented by additional safety data from wider IL-17i clinical trials, supporting that IL-17i–induced SLE remains a rare entity. This is the first such review collating the evidence base of current IL-17i use in SLE and addressing safety and practical implications for its use in SLE.

Despite a limited number of case reports linking the onset of SLE to the initiation of IL-17 inhibitors, these drugs remain a potential therapeutic option for SLE, particularly for cutaneous and articular manifestations. In cases where *de-novo* SLE symptoms developed after starting IL-17 inhibitors, all patients fully recovered upon discontinuation of the drug. This outcome aligns with many cases of drug-induced lupus, where discontinuation of the offending drug and symptomatic management are typically sufficient for recovery (23). However, genetic assessment using Mendelian randomization (MR) was unable to clarify the role of IL-17 in the pathogenesis of SLE, due to challenges such as weak instrument bias and genetic confounding (24).

Furthermore, publication bias may lead to positive reports of IL-17i use in SLE being captured, but in parallel there may be reports not available in the literature. Incomplete follow-up in the reported cases affects the interpretation and significance of the reports, and combined with the few reports, precludes subgroup analysis within this review. The risk of bias amongst those who developed SLE was overall moderate compared to those treated with IL-17i for SLE, which was low. Although heterogeneity was not formally assessed, the case reports had a diverse range, ethnicity pool with a mix of different phenotypes of SLE.

The efficacy of IL-17i for use in SLE remains unclear due to the limited data from published case reports. Amongst the case reports included here, there was significant heterogeneity in the reporting of disease activity, with no standardization or use of classic disease activity metrics or response criteria described. This means that the interpretation of these results for wider subtypes of patients with SLE is challenging, particularly with regards to validating the findings of these studies. Short follow-up periods following treatment and lack of control in terms of concurrent therapies similarly hinder the appraisal of the efficacy of secukinumab in this disease area.

Further limitations fall into safety and the definitions of new-onset SLE. It is unclear from the evidence provided as to whether these are truly paradoxical reactions or episodes of drug-induced lupus. It is difficult to evaluate if this is truly new SLE (i.e., lack of reporting on fulfilment of EULAR/ACR classification criteria (25)) and it is unclear if the paradoxical reaction similar to that seen in TNF/drug-induced lupus is due to lack of reporting of anti-histone antibodies and other markers (26). The definition of remission was heterogeneous amongst the case reports, with the absence of SLEDAI and BILAG scores in the reports (27, 28).

Amongst the limitations directly related to Mendelian randomization, our analysis was limited by the absence of a validated pQTL for IL-17A, meaning we relied on eQTLs that may not accurately reflect circulating IL-17A protein levels and increase susceptibility to genetic confounding. Additionally, the lack of co-localization between IL-17A expression signals and SLE risk reduces confidence that both are driven by the same causal variant, weakening causal inference.

To date, serum IL-17 levels have been shown to correlate with SLE disease activity in a directly proportional fashion. In a study of 36 patients with SLE with a wide variety of phenotypes, IL-17 levels were found to be higher in those with renal and central nervous system disease (29). Animal models have demonstrated similar results with increased IL-17 but also IL-10 signatures in those with increased SLE activity (30). In a further study isolating patients with lupus nephritis alone, serum IL-17 levels were found to be higher with disease activity amongst those with class 3, 4, and 5 nephritis (31).

It is also important to consider that IL-17 belongs to the same immunological axis as IL-12/23, with IL-23 known to have a role in stimulating Th17 cells (32). A previous phase 2 trial of ustekinumab (LOTSUS), an IL-12/23 inhibitor for treating SLE (*n* = 166), showed promise for use in the management of SLE with no safety concerns (33). However, in the phase 3 trial, the interim efficacy data did not demonstrate a significant difference and the trial was discontinued following the analysis due to inefficacy. As is the challenge with many clinical trials in SLE, possible reasons for the failure to meet primary endpoints have been suggested due to small sample sizes, short follow-up durations and placebo response rates (34).

Given the initial positive response from the phase 2 trials on IL-12/23 inhibition in SLE, the question remains as to whether IL-17 inhibition would be of more value in the treatment paradigm for SLE. Serum IL-17 has shown to be present in higher concentrations than IL-12 and 23 in those with SLE (32, 35, 36). With 92 investigational products currently in clinical trials for SLE, IL-17 inhibition remains a potential therapeutic target (37). The current phase 3 SELUNE trial for lupus nephritis (NCT04181762) commenced in 2020 with an estimated 460 participants enrolled, for which the results are due to be published in 2026. The results from this randomized controlled trial will pave the future for whether IL-17 truly has therapeutic potential in SLE.

To date, IL-17i has been primarily used for those with skin and joint disease rather than those with pleural, renal, or SLE affecting the central nervous system (38). In those patients with SLE who are managed with IL-17i, we would suggest some practical points in monitoring with regular serology and urinary protein levels being quantified to assess for worsening disease. The risk of developing SLE from IL-17i exposure based on the literature remains low, with the benefit of using IL-17i to control inflammatory disease and thereby reducing cardiovascular risk with controlled disease, and an improved quality of life equates to IL-17i being a viable option in such selected cases (39)f.

In conclusion, the role of IL-17i in SLE is an area for further investigation (40). As with many targeted therapies in SLE, improvements in scientific research are needed to better understand the role of IL-17 in the pathogenesis of SLE and in which subgroups IL-17i may be effective. Dedicated studies and immunologically stratified trials of clinical efficacy are needed to better understand if there is a role for IL-17i in SLE.

## Data Availability

The original contributions presented in the study are included in the article/supplementary material. Further inquiries can be directed to the corresponding author.
